# Monograph on Puerperal Diseases

**Published:** 1842-01

**Authors:** 


### Art. V.

1. *Monographie der Puerperalkrankheiten.* Von Theodor Helm, Dr. der Medicin, Chirurgie und Geburtshülfe, emerit. Assistenten am Wiener-Gebärhause, &c.—*Zürich*, 1839. 8vo, pp. 152.

*Monograph on Puerperal Diseases.* By Theodore Helm, m.d. &c.—*Zürich*, 1839.

2. *Die Krankheiten der Wöchnerinnen nach den in der k. k. Entbindungs-anstalt und im allgemeinen Krankenhause zu Prag gemachten Beobachtungen.* Von Franz Kiwisch, Ritter v. Rotterau, Doctor der Medicin und Chirurgie, Magister der Geburtshülfe, Assistenten und Secundärarzte an der k. k. Entbindungsanstalt. Erster Theil—*Prag*, 1840. 8vo, pp. 319.

*The Diseases of Women in Childbed, from Observations made at the Lying-in Institution and General Hospital at Prague.* By F. A. Kiwisch, m.d., &c.—*Prague*, 1840.

The above essays on puerperal diseases are valuable as being the results of experience gathered at two of the largest midwifery hospitals upon the Continent, those of Vienna and Prague; Dr. Helm having derived his materials from the former, and Dr. Kiwisch from the latter. As the two works, although on the same subject, are very different, we shall notice them separately, and in the order in which we have transcribed their titles.

1. In applying the epithet "peculiar" to Dr. Helm's work, we believe that we shall gratify the author's feelings much more than if we had made use of a term which was more decidedly complimentary. We regret to say that from its very first page it bears the stamp of an affected eccentricity, which tends not a little to impair its value, and cannot fail to diminish the respect of the reader for his author, and weaken his confidence in him. Dr. Helm has evidently seen much practice, and appears to possess ample materials for writing a valuable book upon the subject, but from a seeming anxiety to produce an effect and astonish his readers, he has endeavoured to bring forward dogmas which his own observations in almost every page disprove, and which are entirely at variance with the best established knowledge of the present day.

From want of clear notions on the subject of fever, Dr. Helm is led to consider post-mortem examinations as the only means of ascertaining the onset, course, and termination of any general disease. All other methods according to him are incorrect, for "they erect a great scaffolding of symptoms about nothing," (Introd. p. xii;) he therefore warns his readers not to expect any minute detail of symptoms, as this will only be required when new forms of disease present themselves.

"One puerperal disease during its progress frequently calls another into action. Thus uterine phlebitis frequently results from septic inflammation of the mucous membrane of the uterus. If gangrene of the vagina and uterus penetrates to the peritoneum it produces peritonitis. Puerperal diseases bring in their train many other diseases. These must not be looked upon as puerperal diseases, for they are neither primary, nor does their appearance or cause present anything peculiar. Thus metastatic lobular pneumonia frequently appears as a consequence of puerperal uterine phlebitis. In inflammation of the brachial vein or other large venous trunks, this species of pneumonia arises and advances in the same manner," &c. (p. 66.)

The author considers that these "secondary diseases," therefore cannot be regarded as puerperal diseases. False reasoning and deductions are evident throughout the whole quotation. Uterine phlebitis and septic inflammation of the lining membrane of the uterus arise from the same cause; they can therefore scarcely be said to produce each other; the same holds good with uterine phlebitis, and the above-mentioned form of pneumonia, nor is it invalidated by the collateral circumstance of inflammation of the brachial vein being also followed occasionally by pneumonia; in both cases of phlebitis the vein has been inflamed by the contact of a morbid animal fluid, which when carried into the circulation has acted as a poison to the blood, and has been followed by one of those violent efforts of the system to throw it off, by inducing inflammation of some internal structure, especially of a mucous or serous surface, which we see in the ordinary experiments of injecting a poison into a vein. We might as well assert that coryza is capable of producing a rubeloid eruption, or sore throat a scarlet rash, because under circumstances of infection the same cause which has produced one symptom has also produced the other.

After the above quotation, the author suddenly comes to the climax of his views, namely, that there is no such thing as puerperal fever, but there are such things as puerperal diseases.

"These are changes which arise from a morbid alteration of organs, and which pathological anatomy alone is capable of revealing to us: in these organic changes consists the essence of the disease," &c. &c. (p. 7.) . . . Not only do not the different puerperal diseases admit of being classed under one common name of puerperal fever, there is not even a single form which answers to this name, and therefore, for this reason also, there is no such a thing as puerperal fever. What can a veritable childbed fever be, but a fever which only appears in puerperal women? but there is not such a fever, and therefore there is no puerperal fever." (p. 9.)

Dr. Helm informs us that

"The puerperal diseases are inflammations of certain organs. (p. 10.) . . . Experience also proves this. So long as minutely injected vessels, the formation of new vessels, flocculent serum, exudation of lymph and pus are considered as the anatomical characters of inflammation, so long are those diseases inflammations which are attended by these appearances." (p. 11.)

We would ask what is the nature of those puerperal diseases which are so rapidly fatal, and which are attended by so few of the above-mentioned changes, and where the extent of these changes seems to be in an inverse ratio to the severity of the attack? Although the author thinks fit to suppress all mention of such a thing as puerperal fever, he informs us that "the puerperal diseases commence with fever, or are accompanied by it; a puerperal patient cannot be declared convalescent until every trace of the fever has disappeared." (p. 13.) Where fever precedes, attends, and terminates an attack of inflammation, and forms so essential and important a feature in the nature of the attack that the patient cannot be deemed in safety "until every trace of the fever has disappeared," we should be hardly justified in denying the appellation of *fever* to this disease, although it may have been attended during its progress with symptoms of local inflammation; it is therefore not a little unfortunate for the author's theory as to the non-existence of puerperal fever that in the severe species of "puerperal diseases," as he calls them, he is obliged to notice fever not only as being a prominent symptom but as *invariably* preceding the symptoms of the local inflammation. But, after all, the matter is rather one of words than anything else, so long as it is not allowed to influence practice.

We willingly allow that in a few isolated places where Dr. Helm's unlucky crotchet (for we can call it nothing else) does not obtrude itself, his observations are sound and practical. As far as is in our power we will do him the justice to quote these, regretting as well as expressing our surprise that they had not led him involuntarily to form clearer and more correct ideas of the nature of the disease.

He recognizes the sporadic origin of puerperal disease as one of its modes of commencement, and very rightly avoids the error into which others have fallen of endeavouring to assign to it only one mode of origin and propagation. No one has expressed this view of the subject more clearly than Dr. Hannay, of Glasgow, and we may be excused quoting from his little essay on Puerperal Fever, as it has been but little noticed although published in 1827: "Of the contagious nature of this disease," says Dr. Hannay, "much diversity of opinion has existed: some have insisted strenuously that it in no case is communicated by the contagious influence carried from one person labouring under it to another; whilst others refer almost every case to contagion as its source. In these extremes, as always happens, I am inclined to think there is at the same time much error and much truth; for I am induced by the facts I shall detail to infer that the disease may at its origin be sporadic and independent of contagion, and so generated become the source where in epidemic rage it may be communicated by contagion to others."

"The endemic origin of puerperal diseases," says Dr. Helm, "may be explained by accumulation and increased intensity of puerperal miasma and puerperal mephites; which last is capable of increasing even to contagion. The increased cutaneous and pulmonary exhalation of women in childbed, the discharge of the lochia mixed with shreds of decidua always more or less decomposed, the activity of the breasts, produce even with the healthiest patients a peculiar taint of the surrounding atmosphere, which is in continual reproduction, and which a delicate or practised sense of smell can instantly detect on entering a room where a puerperal patient is. We propose to call this puerperal miasma. If many individuals who are excreting this miasma are crowded together into one room, it accumulates in intensity and becomes puerperal mephitis, by the action of which either the disposition which is more or less considerable is only increased, or actual puerperal disease is induced." (pp. 15-17.)

In a note to the above observation he adds, "Between miasma, mephitis, and contagion there is only a difference in degree, and this is frequently determined not less by the period of the disease than by the susceptibility of the individual; so that what acts as contagion in one, in another produces but merely a general impression, or none at all." (p. 17.)

His concluding observation on the subject of contagion is a singular one, and seems to be merely for the purpose of maintaining his notion respecting the non-existence of puerperal fever:

"If we are to believe the assertion of various authors, puerperal mephitis has frequently risen to the degree of contagion in lying-in hospitals. But certainly this occurs very rarely, and at any rate can only take place in the most developed cases of the septic species. For my own part I cannot recollect, even in the most virulent epidemics, a single puerperal patient who could be proved to have either given or received contagion." (p. 19.)

We know not what degree of evidence Dr. Helm may require to make him alter his opinion on this subject; his case appears to be one of oblique vision, a disease which has also affected several of the non-contagionists in our own country. The observations of Mr. Moore upon this subject, and the remarks by this Review, (vol. II. p. 488,) in the notice of his work on Puerperal Fever, are worthy of attention. "The facts," says he, "bearing on the contagious nature of puerperal fever are numerous and forcible. Those writers who have espoused the opinion that this fever is simply the effect of local inflammation are of course opposed to the notion of its being contagious; but if the facts recorded cannot be invalidated, their opposition must prove futile." We may also refer our readers to a subsequent part of the present article, where we notice the same subject in Dr. Kiwisch's work. In both the essays now under notice we might be almost induced to suppose that adynamic puerperal fever had never been witnessed by the authors, and that the cases were chiefly of the sthenic or inflammatory character; but the word "septic" is used too frequently by Dr. Helm to permit this supposition, and we must also do him the justice to say that many of his descriptions are too clear and accurate to allow us to entertain a moment's doubt about it. We should have thought that Dr. Helm's excellent observations on puerperal miasma and mephitis would of themselves have been a sufficient ground to establish the possibility of contagion in cases of puerperal fever, or puerperal diseases as he prefers to call them. In his "general treatment of puerperal diseases," he betrays the same inconsistencies. "The pure antiphlogistic method," he says, "is the only one from which success in the majority of cases can be expected. The course of the disease, or its original character, frequently demand the antiseptic treatment, the actual recovery requires the negative treatment in a high degree." (p. 30.) We willingly agree that the antiphlogistic treatment is the most successful, for it is only in the milder attacks that we are able to use it; but are we justified in calling diseases inflammations, the course of which, "or their original character, frequently demand the antiseptic treatment?" The very word "antiseptic" shows that the treatment is to correct or prevent something of the putrid, typhous, or malignant character; and it seems incredible that, with such admissions staring him in the face, the author should persist in calling puerperal diseases inflammations, and denying the existence of puerperal fever.

Dr. Helm's observations on the construction of lying-in hospitals are excellent:

"A lying-in hospital must have a considerable number of rooms for only a few patients; and in order to admit of the changes which are so necessary in the institution, it should have a larger number of rooms than are even necessary for the greatest influx of patients. These rooms must be sufficiently spacious, and capable of being easily warmed and ventilated, and after having been used for some weeks should stand entirely empty for some days, and should be fresh whitened twice a year. The transport from the labour-room into the wards should be without risk, and those who are ill must be separated from the other patients." (p. 31.)

Exactly similar opinions have been expressed by Dr. Ferguson in his valuable work on Puerperal Fever.

Dr. Helm's list of what he calls puerperal diseases is as follows: 1st, inflammation of the veins of the uterus, metrophlebitis puerperalis; 2d, inflammation of the mucous membrane of the uterus, metrophlegmhymenitis puerp.; 3d, inflammation of the ovaries, oophoritis puerp.; 4th, inflammation of the peritoneum, peritonæitis puerp.; 5th, inflammation of the vagina and perineum, koleitis and perinæitis puerp.; 6th, inflammation of the mammary glands, mastitis puerp.; 7th, cutaneous inflammation resembling scarlatina, purpura puerp. (p. 32.) And these are all the diseases to which the author thinks fit to apply the name "puerperal."

"The reader will probably miss puerperal mania, miliaria, and phlegmasia dolens. They are not self-existing diseases, but may be induced by many of those already mentioned. There are no other puerperal diseases, and much as has been talked and written about puerperal typhus, the various puerperal fevers, puerperal meningitis, &c. they do not exist." (p. 33.)

The anatomical characters of the uterine phlebitis are well given:

"The caliber of the inflamed veins is frequently enlarged to the thickness of several goose-quills, and in isolated spots so much so as to admit of the little finger; so that, without close examination, we might be led to the supposition that we had opened an abscess, were not the inner surface of the cavity continuous with that of the vein, and followed its course in both directions. Usually speaking, some veins at the spot where the placenta was attached suppurate first, the other larger ones are plugged with coagula; pus is also frequently found at the beginning of the disease in the veins at the sides of the uterus. But where it has lasted some time most of the veins of the cervix, body, fundus, broad ligaments, fallopian tubes, and even ovaries, are filled with pus and partially inflamed. If the above-mentioned abscess-like dilatations of the veins are situated near to the peritoneal surface of the uterus, they produce a brownish discoloration of it, and on making an incision into the part it will be followed by a free discharge of pus and sanies (Jauche). In many cases the uterus is so traversed in all directions by suppurating veins, that it has the appearance and consistence of a sponge, its substance in every part so altered as to be capable of being easily rubbed down into a dirty paste." (p. 34.)

Dr. Helm, on several occasions, recognizes the possibility of pus becoming mingled with the blood in cases of long-standing phlebitis, but the vitiation of the blood by putrid animal poison never seems to strike him, and were it not for the documents left us by Boer respecting the character of puerperal epidemics at Vienna, and also his admissions in different parts of the work, a superficial reader might also be induced to fancy that Dr. Helm had not met with a case of genuine adynamic fever. At any rate he describes appearances which are by no means common; thus, among the internal and external metastases, the order of frequency with which certain changes take place is by no means the same as is usually observed with us, or that observed by Dr. Kiwisch at Prague.

"Most of the organs of the different cavities are exposed to purulent deposits in uterine phlebitis; they are seen most frequently in the lungs, not so frequently in the spleen or liver, rarely in the brain or in the kidney, and rarest of all in the heart and eye." (p. 39.)

We formerly stated, in our review of Mr. Moore's work on Puerperal Fever, our conviction that uterine phlebitis is produced, generally at least, by the action of an animal poison; in our notice of Dr. Kiwisch's work we shall take the opportunity of stating why it is so frequently preceded by symptoms of adynamic puerperal fever.

"In like manner as the internal organs are subject to such deposits, so are also the external ones; thus they are frequently found in the thyroid gland, still more so in the large salivary glands. Pus is very commonly contained in the different joints of the body, frequently in the symphyses of the pelvis. The metastases to the skin appear either as bullæ of pus beneath the epidermis, or concentrated under the form of infiltrations of the subcutaneous or intermuscular cellular tissue." (p. 41.)

The first two appearances are rare; all the others are well known to be not uncommon results of malignant puerperal fever.

The symptoms of uterine phlebitis, as enumerated by Dr. Helm, show too clearly that in spite of his preconceived notions as to the non-existence of puerperal fever, he cannot help admitting its presence every now and then. We quote it as a very fair picture of commencing puerperal fever:

"Usually on the second or third day after labour, frequently not before the third or fourth week, the patient begins to be feverish; sometimes the attack is preceded by shivering, sometimes not. Sometimes abdominal pain accompanies the fever, and examination points out the uterus as the painful organ. Sometimes the patient is not aware of the pain until the examination has taken place; frequently there is no pain at all: it may, as well as the fever, be very trifling, or it may rise to a great degree of intensity. The patient frequently complains of headach, heat, and thirst; nevertheless it not unfrequently happens that at the beginning of the attack there is not a single symptom besides the feverish pulse, and if the diagnosis be made too soon, it may easily be mistaken for oophoritis, or peritonitis (which can also begin without any pain) and for scarlatina puerp.; yet not for long; although it cannot be denied that not unfrequently phlebitis affords no sign whatever of local suffering, and it is only in the further course of the disease that the diagnosis can be decided." (p. 43.)

There is nothing more in this description, nor in what follows, beyond the ordinary commencement of puerperal fever before the local symptoms have been produced; it may be the peritonitic or gastro-enteric form, as well as phlebitis, according to the part on which, in the language of Dr. Ferguson, the disease is about to spend its chief virulence. Faulty as the author's views are respecting the non-existence of puerperal fever, we can scarcely consider them more erroneous than the opinions of those who affirm puerperal fever to be simply phlebitis, &c.; thus confounding one disease with another; putting the subject in a false light, and involving it in great confusion.

Dr. Helm considers that rigors coming on at rather an advanced stage of the disease are a characteristic mark of uterine phlebitis:

"Patients may continue to have fever from eight to ten days without the presence of any other morbid appearance. All at once the woman is seized with a rigor, followed by a second and third: these confirm the diagnosis; they do not indicate the commencement of suppuration, but show that pus has been carried into the circulation." (p. 45.)

Rigors at this stage of the disease are by no means characteristic of uterine phlebitis, as it is frequently met with where they have never appeared, and vice versâ. A violent rigor on the second or third day after labour, followed by flushing, headach, a quick and usually feeble pulse, which maintains a rate of above 120, is a far more suspicious symptom, for it indicates the incipient vitiation of the circulating current by an animal poison; and although not an invariable symptom, is one of the most frequent, and certainly the earliest warning of incipient puerperal fever, whatever may be the form of "puerperal disease," or local inflammation which may afterwards result.

"The rigors frequently simulate a regular type, although they never acknowledge a periodical regularity; sometimes there are intervals of complete apyrenia, at others one attack runs into the other. During the attacks the various metastases take place, which usually in their extent stand in direct relation with the number and violence of the febrile attacks," (pp. 46-7.)

We agree with Dr. Helm in denying that phlegmasia dolens is inflammation of the crural vein, although we cannot accede to his opinion of its being inflammation of the surrounding cellular tissue. In this respect we agree entirely with Dr. Kiwisch, viz., that it is inflammation and obstruction of the lymphatics usually where they enter the femoral ring. We willingly allow that inflammation of the cellular tissue surrounding the crural vein will in most cases produce a similar condition of the numerous lymphatic trunks which perforate that layer of it (the fascia cribriformis) which fills up the femoral ring, and that as the cellular tissue about the vein is almost necessarily inflamed when the vein itself is, it will be evident that in cases of crural phlebitis we shall also have phlegmasia dolens; but the converse does not hold good, for phlegmasia dolens can distinctly occur without a trace of crural phlebitis.—We shall notice this subject again in our review of Dr. Kiwisch's volume.

Dr. Helm considers that uterine phlebitis may arise in three ways:

"Firstly, the blood which remains in the veins of the uterus where the placenta had been attached, separates into cruor and lymph, and instead of closing each by a solid plug, the latter of these passes into putrilage, and thus excites inflammation of the inner surface of the veins. Secondly, the contact and absorption of the malignant fluid which is secreted during septic inflammation of the mucous membrane of the uterus induces inflammation and suppuration of the uterine veins. Thirdly, the uterine veins frequently become inflamed without our being able to assign one of the above-mentioned sources as the cause; perhaps it may arise from mechanical injury. The separation and expulsion of the last portions of decidua and placental fibres which have remained adhering to the inner wall of the uterus, take place with more or less putrescence in every puerperal patient; by increase of this, septic inflammation of the mucous membrane of the uterus may be induced. As soon as the putrid secretion, which is thus formed, comes in contact with the veins and is absorbed by them, phlebitis instantly commences, only much more rapidly, because the action of the putrilage is much more intense, and the metrophlebitis then also assumes the septic character. Severe contusions of the uterus or vagina, which have become gangrenous, may give rise to this disease. This mode of commencement occurs much less frequently than the first. The third is the rarest of all." (pp. 58-9.)

Dr. Helm's prognosis of uterine phlebitis is very unfavorable, for it is nothing more or less than that of adynamic puerperal fever:

"As a general rule the disease affords but an unfavorable diagnosis, for even in its mildest attacks a large proportion die; in violent epidemics four die out of five, and even nine out of ten. Cessation of the pain, with continuance or even increase of the fever is a very bad symptom. Cessation of the fever and rigors, even if the pain continues, improves the diagnosis greatly. Repeated attacks of the characteristic rigors, as well as any metastases give a bad prognosis, for they indicate the violence and extension of the disease. Lobular pneumonia, mania, puerperal physiognomy, are especially unfavorable. Jaundice and softening of the stomach are the worst symptoms of all." (p. 69.)

Uterine phlebitis uncomplicated with puerperal fever is not so fatal a disease as the author states, if promptly and judiciously attacked; it is only where the circulation has become extensively vitiated before the mouths of the uterine veins had been closed by the process of inflammation that we see uterine phlebitis attended with such fatal results. It is these "violent epidemics," not of venous inflammation but of malignant adynamic puerperal fever, which spread such devastation, especially in lying-in hospitals. We feel confident that every unprejudiced practitioner of any experience will agree with us in saying that the local symptoms, whether of uterine phlebitis or peritonitis (and they are generally complicated with each other), are almost always alleviated or removed by the proper treatment, if quickly and energetically applied; the pain ceases, the incipient tympanites subsides, the patient declares herself comfortable and well; but the mark of a vitiated circulation, and therefore of danger, still remains, viz. a quick small pulse, of at least 120, and frequently much more in the minute; the hippocratic change in the features, which the author has denominated puerperal physiognomy, now gradually steals on, collapse becomes more and more apparent, and thus she sinks. In the worst cases there is this appearance at the outset of the disease, for it is a state of collapse from the very beginning.

In giving the results of dissection in cases of puerperal peritonitis, Dr. Helm states that, besides the inflamed state of the peritoneum there is frequently "contemporaneous pleuritis, pericarditis, meningitis, gangrene of the lung, softening of the stomach, caries of the pubic cartilage, abscesses beneath the skin and beneath the muscles; lastly, serous or purulent infiltration of the subcutaneous or intermuscular cellular tissue." (p. 100.) These, and many other more-commonly-seen affections are most truly contemporaneous with the peritonitis, for they arise from the same cause, viz. puerperal fever.

"Softening of the stomach is characterized by its mucous lining being converted into a blackish-brown gelatinous paste; in the worst forms all the coats of the stomach are implicated. It is usually seen in the large curvature; its walls are frequently perforated. The softening frequently extends not only over the larger portion of the stomach, but the diaphragm, œsophagus, even the pleura and lungs, implicated in the process of softening, are converted into the same mass and perforated. That the various ruptures of the stomach, diaphragm, and œsophagus, have not occurred after death or during the last moments of life, is proved by the incipient inflammatory reaction about the above-mentioned spots, as far as the coffee-coloured fluid and softening have extended in the thoracic and abdominal cavities." (p. 101.)

Dr. Helm's summary of the effects of puerperal peritonitis, when not immediately fatal, are interesting:

"If death does not quickly ensue after effusion has taken place in cases of puerperal peritonitis, either recovery quickly takes place by absorption, or the disease takes on a chronic form. In the latter case absorption takes place either with continued fever and no metastases, or with the formation of metastases; the effusion makes its way through the abdominal cavity; the lymphatic exudation is converted into purulent or tubercular deposit, finally dropsy and obstructed nutrition, or death from exhaustion." (p. 43.)

The author's treatment in puerperal peritonitis where it is not, as he calls it, "septic," consists of general and local bleeding, according to the severity of the attack, with ice fomentations and mercury in large doses; "the septic form of puerperal peritonitis," he says, "defies all treatment."

"Nothing is capable of relieving pain and fever so quickly (the former almost instantly), as ice fomentations spread over the abdomen, but they must be diligently changed and continued long enough, viz. two, three, or more days." (p. 141.)

In recommending the use of mercury he advises that it should be freely rubbed in, both on the abdomen and thighs, and considers that it has frequently been attended by the best results, a fact which we can also confirm. He also praises the application of ice very highly in threatening milk abscess from inflammation, *mastitis puerperalis,* which forms the subject of his last chapter.

"Early and frequently repeated application of the child to the breast, spare diet and caution against cold, are the best prophylactic means of treatment in this disease. But if it has nevertheless come on, the surest means of avoiding or stopping the suppuration which usually comes on so quickly is to apply ice-fomentations to the inflamed breast for at least twenty-four hours. We need not fear the effects of this application, and it is astonishing how rapidly and favorably it acts." (p. 145.)

We have passed over many vague and inconsistent opinions without comment, and can only regret that their frequent or almost constant recurrence have marred the value of what might have otherwise been an excellent essay on the subject; it is only surprising that the author has been able to record such facts and make such observations, and yet with every semblance of candour persist in the assertion that there is no such a thing as puerperal fever. Dr. Helm's work is a good lesson to those who look upon pathology as synonymous with morbid anatomy. Advancing knowledge, increasing experience, and unprejudiced observation have, we trust, long ere this, convinced every open and candid mind that the scalpel is *not* the only mode of investigating disease.

II. Dr. Kiwisch's field of observation has been no less extensive than Dr. Helm's. Although less known than that of Vienna, the Lying-in Hospital at Prague holds a distinguished rank among those of the continent, nearly 1800 women being annually delivered within its walls. This picturesque city contains several other admirable medical institutions, especially an extensive general hospital, and an asylum for lunatics more recently established. The work of Dr. Kiwisch is dedicated to Professor v. Krombholz, a distinguished ornament of the "Prager Universität." The wards under his care present a combination of advantages for students as well as patients, which we have seldom seen equalled, and perhaps never surpassed. Their admirable form and arrangements for ventilation, the scrupulous attention to neatness and cleanliness, and above all, a strict system of clinical instruction carried on in Latin at the bed-side, struck us very forcibly on visiting Prague some years ago. Neither have we forgotten the noble bearing and highly intellectual countenance of the Professor himself, nor his affable courteousness to strangers; and we can assure those of our medical brethren who are about to travel on the continent, that a visit to the hospital, &c. at Prague will well repay them for so long a journey. With regard to the Lying-in Hospital itself, we will let the author speak for himself:

"The situation of the institution is one of the highest parts of the town, freely exposed to the fresh air which comes over extensive gardens on three sides of it. The wards are sufficiently roomy and light, they are whitened every year, and are constantly ventilated with the necessary caution. Twelve large and five smaller wards are appropriated for the reception of women in labour, and those in childbed. The number of patients in the large wards never exceeds from 5 to 7." (p. 56.)

The author of this volume is evidently a man of great experience; at any rate he has had ample means for becoming so, being assistant-physician to the above establishment, and having also seen the extensive practice of the Lying-in Hospital at Vienna. He appears also to be a hard-working well-read man, and has premised a very elaborate and valuable list, chronologically arranged, of every work which has treated upon puerperal diseases from the time of Hippocrates down to the volume by Dr. Helm, which we have just given an account of. But with all this show of learning and experience, the merits of which we are fully willing to appreciate, he appears unable to form any general or enlarged idea of the subject, or to divest his mind of those trammels of reasoning, into which he has been led by the too common error of mistaking the anatomy of a disease for its pathology. On this point he and Dr. Helm are of accord.

Dr. Kiwisch considers that puerperal fever invariably arises from epidemic influence, and that its sporadic occurrence is very improbable; an opinion to which we can by no means subscribe, for ample evidence and experience have long since convinced us that it frequently does exist sporadically, and this even in its worst forms: we might also add that it is occasionally seen to exist *endemically*, among the patients of certain practitioners who are too eager to investigate the anatomical characters of the disease, which thus becomes propagated not by epidemic causes, but by the more direct and simple process of contagion. He exclaims against the French authors who, like Dr. Helm, would reject the term "puerperal fever" altogether, and call it inflammation of the different organs which may happen to have been attacked; and yet with singular inconsistency, when he speaks of the different forms of puerperal fever, he actually brings them under the three following heads: 1, Peritonitis; 2, Inflammation of vessels, phlebitis, and lymphangioitis; 3, Inflammation of the mucous membrane of the uterus, metrhymenitis, endometritis.

We are far from wishing to deny the all-pervading influence of epidemical causes, and quote the following passage not only for the sake of concurring thus far with the author, but also to show some of the many variations in type which are exhibited by this disease:

"Puerperal fever cases have constantly been observed in groups, not merely in the large lying-in hospitals, but also elsewhere; and the great epidemics during the last few years at our hospital, namely, in 1832-4-5 and 1839, agree more or less with similar epidemics over the whole European continent. The effects of the miasmatic influence is frequently so great as to overcome the greatest variety of individual peculiarity, and stamp the same type upon every case, producing a remarkable similarity in the progress of the various cases of puerperal fever at the time. Thus in proof of what has now been stated, I saw in the course of 8 days in two patients at the Lying-in Institution, and in a third at the general hospital, the metastatic ophthalmia which is so rare and which had not been observed for some time before, although the epidemic had prevailed. In like manner no cases of miliary eruption appeared during the first half of the epidemic of 1839, whereas in autumn, in the course of a few days, it appeared so generally both in the town as well as in the institution, that almost every puerperal patient was bathed in watery perspiration. In the spring months of 1839, abscesses of the vagina, which had not been seen for a long time or since, attended nearly all the cases of puerperal fever." (p. 22.)

We especially wish to direct the attention of our readers to the last sentence of this quotation, as it refers to a feature in puerperal fever which has not been commonly observed in this country. We willingly agree in the opinion that not only is the disposition of the female system to disease considerably increased by the different changes of the uterus as in menstruation and childbed, but also that it is more susceptible to epidemic influence at those times; and the fact which Professor Sebastian has remarked that women are peculiarly liable to be attacked by marsh fever when menstruating, is interesting in proof of this assertion. That the system is equally susceptible at the time of conception is, we think, very doubtful; indeed, from the numerous facts collected by Burdach, there is every reason to suppose that it is just the reverse, it being found that animals not only exhibit a much greater tenacity of life at these periods of excitement, but are unaffected by agents (poisons, &c.) which under other circumstances would speedily prove destructive to them.

Although the author has recorded his opinion in favour of the *inflammatory* origin of puerperal fever by the arrangement which he has adopted of its different forms, he nevertheless alludes to the opinion that the blood, which is "the origin of all organic action," undergoes a peculiar change in this disease,—he acknowledges that many facts prove that puerperal fever is a disease of the blood, but appears unable to make a single deduction from this view of any value; he becomes bewildered among the different *results* of puerperal fever, namely, peritonitis, exudation of coagulable lymph, phlebitis, &c. which he mistakes for the disease itself. This error is most conspicuous in his enumerations of the various species, each of which is characterized as being some peculiar form of inflammation. We have long since shown, and our opinion has been confirmed by Dr. Ferguson in his recent work on this subject, that puerperal fever is *not* identical with or dependent upon inflammation, but that it depends upon a vitiated condition of the circulating current. The author now and then gets upon the right track, but soon wanders from it, blinded by theory and erroneous deductions from morbid anatomy. He attempts to disprove Cruveilhier's opinion that the increase of puerperal fever cases, both in number and intensity, is in exact proportion to the number of lying-in patients, a view of considerable value, and which proves the identity of origin between this disease and hospital typhus, &c. Mere numerical comparisons between the number of patients and fever cases give us no accurate conclusions, as the occurrence now and then of puerperal epidemics will be quite sufficient to destroy all means of estimating the real proportion; we therefore do not quote the following numbers in proof of M. Kiwisch's views, but as furnishing some interesting statistical data of the Lying-in Hospital at Prague.

| | | | |
|---|---|---|---|
| In 1831, | 7 | patients died out of | 1161 |
| 1832, | 14 | .. | 1285 |
| 1833, | 36 | .. | 1297 |
| 1834, | 87 | .. | 1343 |
| 1835, | 63 | .. | 1293 |
| 1836, | 5 | .. | 1449 |
| 1837, | 7 | .. | 1455 |
| 1838, | 11 | .. | 1694 |
| 1839, (to the end of Nov.) | 37 | | 1597 |

The author considers that from 3 to 7 died yearly from other diseases than puerperal fever.

He takes a very sensible view of the causes which render patients in lying-in hospitals peculiarly liable to this disease, and justly observes that the number of patients attacked and of fatal cases must ever be greater in hospitals than in private practice, since in the former they are chiefly of the lowest classes, who have been exposed to all sorts of deprivations, depressing passions, &c. and in whom the health is more or less broken down. We cannot certainly accuse him of over-consistent opinions, when, after opposing Cruveilhier's views above mentioned, he terminates the paragraph by the following correct observation: " It must not be overlooked that a number of persons lying together, freely exposed to effluvia in epidemic diseases, easily render the character of these diseases more dangerous." (p. 40.) We were rather surprised to find that he denies the *contagious* nature of puerperal fever, and declares that although the patients who were affected were not always separated from the healthy ones at his hospital, he never observed a case where one patient caught the disease from her neighbour. We would, however, much rather form our opinions of its contagious or non-contagious nature on the answer given by the author as to what was the fate of each patient who was delivered by him after making a post-mortem examination of the fatal fever cases in the hospital? A similar erroneous opinion seems to prevail at the Vienna hospital, as far as we can judge from an observation of Dr. Helm.

It is to the British practitioner that we are indebted for strongly insisting upon this important and dangerous character of puerperal fever. None has illustrated it more strikingly than the late Dr. Gooch, (on the Diseases of Females, p. 4,) and it is to those who deem it necessary to examine minutely after death every case of fatal puerperal fever, that we would most earnestly impress on their minds the following observation of Dr. Rigby: "Where a practitioner has been engaged in the post-mortem examination of a case of puerperal fever, we do not hesitate to declare it highly unsafe for him to attend a case of labour for some days afterwards. The peculiar-smelling effluvia which arises from the body of a patient during life is quite, in our opinion, sufficient to infect the clothes; and every one who has made a minute dissection of the abdominal viscera, especially in fatal cases of puerperal fever, knows full well that it is almost impossible to remove the smell from the hands for many hours, even with the aid of repeated washing; it must be therefore self-evident that, under such circumstances, it would be almost criminal to expose a lying-in patient to such a risk." (Library of Med., vol. vi. p. 292).

We have before stated that Dr. Kiwisch appears to be a man of observation, although he makes but little use of his observations in the way of deduction.

"Operations," he says, "which in the absence of an epidemic are not attended with even the slightest unfavorable results, during its presence are frequently fatal; indeed we may almost certainly calculate upon an attack of puerperal fever where we have had a difficult case of turning, or separation of the placenta during a severe epidemic. In the same way we see the formation of gangrenous abscesses after easy forceps cases during an epidemic, which would not have occurred at a more favorable time. If such results were to deter us from operating, we should only expose the patient to still greater danger; since, where the uterus has been overstrained during labour, it is more susceptible of disease, and hence it is advisable rather to operate early." (p. 43.)

But why should it be the fact that it is dangerous during a puerperal epidemic to repeat the vaginal examination at all frequently, and that the introduction of the hand, whether for turning or removing the placenta, &c. is almost certain of being followed by a severe attack of the malady? Simply because it is what the author doubts it to be, *contagious.* Within a week from the time of our writing these words, a fatal proof of this fact has occurred to us. A young practitioner, contrary to advice, examined the body of a patient who had died from puerperal fever; there was no epidemic at the time, the case appeared to be purely sporadic: he delivered three other women shortly afterwards; they all died with puerperal fever, the symptoms of which broke out very soon after labour; the patients of his colleague did well, except one, where he assisted to remove some coagula from the uterus; she was attacked in the same manner as those whom he had attended and died also: we trust that this fact alone will for ever silence such doubts, and stamp the well-merited epithet of "*criminal,*" as above quoted upon such attempts.

Although the well-known fact of dangerous attacks of puerperal fever being seen, especially after cases of hemorrhage, stares him in the face as an argument against the inflammatory origin of the disease, the author endeavours to explain it away by some far-fetched reasons, quite forgetful or ignorant of the fact that vitiation of the blood has arisen from the presence of putrid coagula and lochia retained in the passages.

Although we quite agree with the author that those cases of puerperal fever which appear soonest after labour are usually the worst, we beg to differ from him most widely when he considers that "every lying-in woman who has continued quite well during the first ten or fourteen days after her confinement is perfectly safe against an attack of puerperal fever," (p. 49;) as we have repeatedly seen severe cases of the disease commencing from the tenth to the fourteenth day after delivery, although we willingly own that the majority commence at a much earlier period. Neither can we agree that "the usual seat of the critical discharges is the skin," (p. 51,) for it is by no means uncommon to see profuse perspirations during the early attacks of puerperal fever, especially of the adynamic sort, without any diminution of the quickness of the pulse or relief to the patient, in some of the milder and more inflammatory forms; a general diaphoresis is favorable, but we consider the bowels, the uterus, the liver, and the kidneys, much more capable of affording what may be called critical discharges in this disease.

In his chapter on the treatment of puerperal fever, Dr. Kiwisch considers it under the eight following heads:—1, general bleeding; 2, topical bleeding; 3, purgatives; 4, emetics; 5, mercurials; 6, oil of turpentine; 7, ice; 8, vaginal injections. We gain however but little from his observations; for, although he appears to have read extensively upon the subject, he does not seem aware that different authors have described a considerable variety of affections under the same head of puerperal fever, and hence leaves the inexperienced part of his readers in utter confusion and uncertainty as to what line of treatment ought to be adopted. He attempts to compare the practice of those who did not bleed with that of those who bled freely, and considers that the results of the former were invariably much more unfavorable. He endeavours to explain this variance of practice partly from the fact "that there really are forms of puerperal fever which either require slight bleedings or none at all," (p. 61;) but he does not tell us that there are forms of puerperal fever which do not *admit* of bleeding, which are of a low, adynamic, malignant character which puts it out of the question; and that this alone is the reason (and a sufficient one too) why those epidemics in which bleeding has been found inadmissible have proved so fatal. He does not tell us that the very fact of this remedy being indicated is the best possible proof that the disease is not appearing under its most deadly form, but from his reading and experience he considers "that general bloodletting will always remain an essential in the treatment of puerperal fever." From this and other observations, and also from his arrangement of its different forms as so many species of inflammation, we are led to the conclusion that the cases which he has witnessed were chiefly of this character. He is cautious in the use of purgatives, but considers "that mercury, when given so as to induce symptoms of salivation, is one of the best remedies in most puerperal fevers, and that it can be given with much advantage in the metastatic forms of inflammation, and even in the adynamic stage." (p. 66.) The correctness of the last observation may be very fairly doubted, and if ever the calomel is to be of use in this stage, it is rather in a single dose of eight or ten grains, than given in smaller and repeated doses combined with opium. "Small doses," as Dr. Ferguson justly observes on the treatment of the gastro-enteric form of puerperal fever, "create purging, pain, and irritation, while the full dose produces one to six large pultaceous stools; after which the tongue is cleaned, rendered less red, and more moist, and the pulse usually falls. These stools, when examined, appear to contain the fecal matter suspended in large quantities of mucus and greenish bile, as if the turgid capillaries of the irritated intestinal canal and liver had been freed from their load."*

Dr. Kiwisch gives a short account of the different trials which have been made with oil of turpentine, and gives a quotation from Dr. Michaelis's Observations on the Use of Ice, of which we gave an abstract some time ago.† On the use of opiates, except when given with calomel to promote salivation, Dr. Kiwisch is silent, as also upon the subject of saline medicines; indeed most of his observations on the treatment are far inferior to the excellent ones in Mr. Moore's able work, and still more so in every respect to the clear and practical views of Dr. Ferguson. The sound reasoning and deductions of the authors just named contrast

* Essays on the most Important Diseases of Women, by R. Ferguson, M.D. p. 159.
† Brit. and For. Med. Review. Oct. 1837.

strikingly with the vague no-opinions of the present work, which we shall therefore pass over.

The author's description of the manner in which effusion of lymph takes place in peritonitis is worthy of attention; indeed, he appears to have accurately investigated the "anatomical characters" of the disease; and, like many others, evidently supposes that in so doing he has been describing its pathology:

"At the commencement of the disease the exudation is clear, lymphatic, reddish, and covers the peritoneum with a uniform slimy layer; when it has been secreted a few hours, and has accumulated in larger quantities, it collects in the lower parts, becomes thick, flocculent, and separates into two layers, a serous and a flocculent one; the latter deposits itself upon the abdominal organs, namely, the liver, the spleen, the uterus, the intestines, and ovaries; it is partly absorbed, and forms upon these organs membranous coverings, which at first are soft and can easily be wiped off, and afterwards become tough and cellular, forming firm adhesions between the folds of peritoneum, or become liquid and purulent, whilst the serous portion is more or less absorbed. In those parts where the flocculent portion is collected in considerable quantities, it becomes more homogeneous, and assumes a purulent appearance." (p. 74.)

Dr. Kiwisch has given an elaborate chemical analysis of the exudations in puerperal fever, which our limits forbid us to insert, but we must be allowed to quote his observations on the acid or alkaline condition of these fluids:

"The chemical characters of the exudations vary in different individuals, as indeed is usually the case with morbid secretions; this is not only confirmed by the different results of the analyses which have been made, but also from the fact that even a cursory examination of the fluid discovers different properties. Even the test of vegetable colours gives different results; thus Jacquin Chaussier found it alkaline, Busch neutral, Kastner acid, and according to my own observations it was sometimes one sometimes the other; nevertheless, I have reason to think, that when it is quite fresh it is always slightly acid, for on puncturing the abdomen of a patient suffering under the disease, the effused fluid which escaped was remarkably acid." (p. 89.)

It is interesting to observe how far the characters of puerperal fever differ at Prague from those which are ordinarily observed in London, situated as these two cities are, so far apart, one in the eastern, the other in the western part of Europe. In the work before us we find here and there appearances described which are not commonly, or perhaps scarcely ever, met with in this country; it will therefore be necessary for us to mention them, that they may be recognized should they ever occur here in any future epidemics. Thus, for instance, in general peritonitic exudation and enormous tympanitis, the intestinal canal appears to become so paralysed as to be followed by results not often witnessed in this country. There is "frequently an enormous accumulation of fluid matters in the intestinal canal which continues obstinately constipated till the last moment; in this case it usually comes to vomiting of every possible contents of the bowels, even to the very masses of fæces." (p. 94.)

Another effect of extensive effusion of lymph, &c. into the abdominal cavity is the formation of large sacs or cells, by the intestines, &c. adhering together, filled with purulent matter.

"These sacculated accumulations of pus compress the adjacent organs, and frequently produce considerable cavities, as, for instance, in the uterus, liver, &c. where in a cursory examination they might easily be mistaken for cavities of these organs. (p. 75.) . . . . If the exudation has become sacculated, if it has not attained to any considerable extent, and the constitution of the patient still possesses strength, recovery follows pretty quickly; on the other hand, if the collection of purulent matter be very large, it usually becomes very troublesome from the pressure which it exerts on the adjacent organs, from the sensation of tension and fluctuation, and recurring attacks of violent pain. Not unfrequently lasting functional derangements of the intestines are produced by the great pressure and numerous adhesions of the abdominal viscera, and sterility will result from the adhesion and dislocation of the tubes and ovaries." (p. 99.)

Modifications of this latter condition are occasionally seen during a lingering convalescence from severe puerperal peritonitis in this country, and not unfrequently after a protracted struggle prove ultimately fatal. In some the recovery from the disease seems nearly complete; a little more tone and strength seem all that is wanting to restore the patient to perfect health; but this happy end is not attained; the chylopoietic functions struggle on imperfectly, and only with the constant aid of medicine: at length marasmus sets in and the patient sinks. In others the recovery has been more complete, although the patient has continued frequently subject to deranged bowels, &c. with abdominal pain, the result probably of adhesions from the lymph which was effused during the attack.

Under the "anatomical characters" of what the author calls "*Lymphangioitis,*" we find morbid appearances described which are certainly but little met with in this country. Having mentioned the ordinary changes which are observed in inflammation of the uterine lymphatics, the author alludes to the unusual degree of dilatation which they occasionally undergo. In places they are as large as a nut, and being filled with purulent matter, are usually taken for abscesses in the substance of the uterus.

"Most commonly we find the lymphatics inflamed on the sides of the cervix and body of the uterus, forming hard conglomerate masses, frequently as big as a hen's egg; on cutting into them at the beginning of the inflammation, they are found to contain a firm white mass of exudation, which is either contained in the lymphatics, which are not much dilated or thickened, or which is partially infiltrated into the surrounding cellular tissue: it afterwards solidifies and grows yellower, the lymphatics continue to dilate more until they attain the size above mentioned, and look like so many isolated abscesses." (p. 107.)

We cannot admit that puerperal uterine phlebitis (metrophlebitis) is necessarilly produced by epidemic influence, as experience has long since amply convinced us that this affection frequently, we might even say usually, arises from direct contact of morbid animal matter with the inner surface of the uterine veins. Under such circumstances, the inflammation of the veins cannot be looked upon as the cause of the vitiated state of the blood, but as a prior, or perhaps contemporaneous, effect of the absorption, imbibition, or, at any rate, introduction of morbid or putrid animal matter into them, producing by its contact a state of phlebitis. In many cases their uterine portion becomes quickly impervious from swelling, &c. thus confining the mischief to the local affection; in others, the rapid advance of adynamic typhoid symptoms show that the circulating current has also become affected.

"Piorry considers that the formation of pus in the veins may take place in three ways: by absorption; by the mechanical entrance of pus, which has been poured upon raw surfaces which are in immediate connexion with open venous mouths; and thirdly, by the exudation of pus upon the inner surface of the inflamed vessel." (p. 114.)

The author expresses it as his own conviction that when pus is found in the vessels it is not the result of absorption; and in the majority of cases we would agree with him, inasmuch as the cavity of the uterus rarely contains any quantity of pus after labour, but a mixture of pus, lochia, half-dissolved coagula, and other fibrinous matter, &c. forming a "*putrilage*," which most assuredly can never remain long in contact with the mouths of the uterine veins without producing dangerous consequences. It is useless for the author to deny that these effects can be thus produced, upon the assumption that the uterus is well contracted, and therefore effectually closes these openings; but supposing the uterus is *not* well contracted, as is frequently the case, is there still to be no danger of the *putrilage* passing into the venous openings of the uterine cavity? Does the uterus in any case remain in one continued state of firm contraction from the moment that labour is over? Does it not slowly and gradually increase in size and diminish in hardness until the second or third day, and then subsides, until it at length resumes the ordinary characters of the unimpregnated state? and does not the quantity of the lochia bear a close relation to the size of the uterus during the first few days after labour? If such were the permanent state of firm uterine contraction after labour, the secretion of lochia would be as rare an occurrence as the absorption of putrilage.

The author's description of the metastatic inflammations which occur in puerperal fever is very good; we cannot agree with him in assigning these local attacks to the state of phlebitis, but to the more general cause, namely, the vitiated state of the circulating current.

"The deposits of purulent matter in the cellular tissue are capable of attaining an enormous extent, and frequently occupy half an extremity. The cellular tissue is frequently extensively destroyed, the adjacent structures more or less implicated in the inflammatory process softened or having undergone a fatty degeneration. The purulent infiltrations are most frequently found in the broad ligaments, in the inguinal, sacral, or other pelvic regions; the deeper they are situated, the greater will be the difficulty in finding their way through the neighbouring parts; at the anterior wall of the abdomen they burst through at the umbilical or inguinal region; in the sacral region I have twice seen them make their way through by the side of the spinous processes of the sacrum, or a considerable quantity of purulent matter had been discharged by the labia. Not unfrequently these abscesses form in the inner surface of the thigh, and are seated either superficially beneath the fascia, or deep among the muscles, and are frequently confounded with phlegmatia dolens. Where the general symptoms assume a typhoid character, the abscesses are filled with putrid matter, and the surrounding soft parts are liable to pass into gangrenous destruction." (p. 131.)

The following observations, it is true, belong more strictly to phlebitis, and present appearances not often met with in this country:

"In most cases where the metastatic pneumonia was violent, I found the pulmonary arteries of the diseased lobe usually inflamed as far as their point of exit from the pulmonary tissue, and filled with adherent lymph and pus." (p. 134.)

The author supposes that the above-mentioned appearances in the pulmonary arteries were produced by extension of phlebitis along the whole tract of venous canal from the uterus to the lungs, an explanation which is quite untenable, and implies an extent of venous inflammation, not to mention a similar state of the inner surface of the right ventricle of the heart, which is scarcely ever met with when the whole structure of the lung has been inflamed, and with it the venous trunks. Pneumonia is not a common attendant upon or modification of puerperal fever, at least as it has usually appeared in London; we have, however, seen two cases recently where it manifested itself, and in the epidemic puerperal fever witnessed by Dr. Kiwisch at Prague it seems to have been of frequent occurrence.

We agree with the author in placing the spleen first in the list of organs which are most frequently found diseased; the liver and the kidneys follow. He describes the organic changes, commencing in the form of lobular inflammation, in spots from the size of a pin's head to that of a walnut, sometimes connected, sometimes very numerous (more than a hundred), superficial or deep.

"The organ attacked is usually found in a state of great congestion; the exudations of lymph have great disposition to dissolve into putrilage, and the parenchyma itself to soften; the structure of the spleen before it comes to the formation of pus frequently breaks down into a red pulp, forming a fluctuating mass within its capsule." (p. 135.)

The effects of puerperal metastatic inflammation in the brain are interesting, the more so as they are not commonly observed:

"The metastatic inflammation of the brain was observed to be of considerable extent, namely, from the size of a pea to that of a walnut . . . . The inflammation here produces at first congestion, with softening of the brain and lymphatic infiltration; the formation of matter comes on afterwards. The purulent deposit is usually surrounded with softened cerebral substance. In one case where the brain was attacked it appeared under the form of pure cerebrophlebitis, for the finest vessels of the inflamed spot (which was as large as a walnut) contained firm plugs of coagulable lymph, which could be traced in a continuous course into the larger vessels, and even extended to the longitudinal sinus: this, as also the lateral sinus to its opening into the jugular vein, was thickened and distended with firm lymph, which in the middle presented a purulent and dissolved appearance." (p. 136.)

The author's description of the inflammation which occasionally attacks the eye in puerperal fever is very minute, but it does not present exactly the same characters which have usually been met with in England; the ring round the edge of the cornea which makes it so closely resemble the characters of common rheumatic or arthritic inflammation, does not appear to have been present in the cases observed at Prague:

"The metastatic ophthalmia exhibits various changes in the structure of the eye according to the degree of its progress: at first there is only swelling with injected vessels, a state which can increase even to extravasation of blood in the sclerotic and conjunctiva, with severe congestion of the vessels in the other parts of the eye, especially of the choroid and of the iris, which becomes discoloured. Lymphatic exudations soon form partly on the surface of the inner linings of the eye, partly between their layers, by which they are thickened, and the choroid, as also the uvea and corpus ciliare, become partially or entirely discoloured: the tunica vitrea, the capsule of the lens, and the structures of the anterior chamber become similarly affected; flocculent exudations of lymph take place in the canal of Petit, in the fluid of the vitreous humour, in the lens, in the chambers of the aqueous humour, and in the cornea. As the disease proceeds, the various structures of the eye become partially softened, disorganized, and the effused matter becomes purulent or decomposed (*jauchig*), perforates the lamina of the cornea, or by destroying the connexion of the sclerotic with the cornea finds a vent externally." (p. 137.)

The characteristics of these metastatic inflammations, as the author calls them, in the muscles are much the same as those observed in this country, but we must reject the notion of their being "in consequence of phlebitis:" our reasons for so doing have been already given. He has described cases where destruction of inter-osseous cartilages has taken place to an extent which we have never witnessed, nor do we recollect to have seen any similar instances recorded by English observers:

"The destruction of cartilage in the synchondroses of the pelvis is a process which differs little from that which takes place in the muscles, except it may be remarked that the accumulation of pus often attains an incredible extent, so that a collection of purulent matter frequently forms between the symphysis ossium pubis as large as a man's fist. Upon examination the cartilages are usually found to be entirely destroyed, and the adjacent bones carious." (p. 138.)

The changes in the skin and mucous membranes, which Dr. Kiwisch has observed, are also very remarkable, and equally uncommon in English practice:

"The metastatic deposits in the skin sometimes appear under the form of boils, sometimes of pustules, which are not unlike those of smallpox, or form large purulent bullæ, which are only covered by epidermis. Between the coats of the intestines under the mucous membrane, collections of purulent matter are found in immense numbers, from the size of a pea to that of a dollar, by which the mucous membrane is raised into fluctuating elevations and irregularities; in like manner small abscesses are found under that of the pharynx and œsophagus." (p. 139.)

The author's diagnosis of phlebitis is very unsatisfactory, and tends to confirm the observation which we have already made, that he has confounded the cause with its effects; he appears unable to realize the fact that the local inflammations which attend puerperal fever are the results of this disease, *not* the disease itself; and from this too common error he is led into still greater ones respecting the symptoms. Our limits will not allow us to combat his assertion that, although the symptoms of phlebitis in superficial structures are sufficiently distinct, we possess no appreciable diagnostic marks of this disease in the uterus, as it would require our entering upon a detailed enumeration of its symptoms.

In describing the results of phlebitis, Dr. Kiwisch offers some excellent remarks on the erroneous views of some author, in endeavouring to confound or identify phlebitis with phlegmatia dolens:

"Œdematous swelling, either of the corresponding extremity or of the lower half of the trunk, may follow obliteration of the femoral or pelvic veins, or of the cava ascendens. This œdema extends from the periphery towards the obliterated trunk, is without pain or change of colour or temperature, easily yields to the pressure of the finger, and is frequently mistaken; it is the more easily confounded with phlegmatia alba dolens, because the course of the inflamed vein is usually very tender; hence we find that, even with distinguished observers, phlebitis is mentioned as the proximate cause of phlegmatia dolens; and Dr. R. Lee considers this latter disease as synonymous with crural phlebitis." (p. 145.)

His observations on the mania and other cerebral affections which sometimes accompany puerperal fever are very excellent, and we quite agree with him in attributing these local attacks to the generally diseased condition of the circulating current; but, as we have already stated, we cannot attribute this latter state to the effects of phlebitis in every case, or even in the majority of such cases. His account of those cases where the disease attacks the heart is equally good, and as they are not of common occurrence we may be permitted to quote him on this subject:

"The metastatic carditis, with deposition of purulent matter in the muscular substance of the heart, is scarcely capable of being ascertained during the patient's life; the metastatic inflammation of the valves (endocarditis), on the other hand, is more distinguished by its symptoms. I saw this latter form of disease repeatedly during the last epidemic; twice in patients who had previously suffered from endocarditis, and who probably were therefore peculiarly liable to be attacked. The disease in these cases manifested itself by violent vascular excitement, strong palpitation, during which upon examination the heart was felt beating powerfully over a surface of many square inches. At the same time the sounds of the heart were more or less dull upon auscultation, running into each other, and indistinct, from more or less sound of *frottement.* No other symptoms elsewhere were perceptible. The course of the disease was very violent, and the patients all sank very rapidly." (p. 158.)

Dr. Kiwisch notices the variety of opinion which exists respecting the nature of phlegmatia dolens, viz. that one party considers it to be phlebitis, others lymphangioitis, or inflammation of the lymphatics; and very justly, as we conceive, declares himself in favour of the latter opinion. In alluding to the other he observes:

"The opinion of these authors was necessarily soon overthrown by the results of pathological anatomy, for many authors of modern times, as well as myself, have found the veins perfectly healthy in cases of the most strongly-marked phlegmatia, and it remains to prove the other opinion which considers inflammation of the lymphatic vessels to be the cause. ...We always find in the acute œdema of puerperal women swelling and pain of the lymphatic glands and vessels, also in the vicinity of the affected part; and even in the post-mortem examinations which have been reported by those who consider that it is phlebitis, we usually find considerable changes of the lymphatic system mentioned. ....When we bear in mind that the metastatic inflammations in phlebitis run an entirely different course, inasmuch as they are usually combined with violent inflammatory redness of the skin and rapid formation of pus, whereas phlegmatia is usually characterized by the white colour of the skin, the formation of serum, and tedious course; it is still more improbable that phlebitis has any share in this disease, and if it be combined with phlegmatia it must be looked upon as an accidental complication, in the same manner as inflammation of the cellular tissue and muscles, which last Hull considers to be the chief seat of the disease in question. If we also consider the course of lymphangioitis in those who are not puerperal patients, we find that wherever the disease is at all intense, acute œdema (phlegmatia) is sure to accompany it. Piorry (Traité de Diagnostic et de Semeiologie, § 6,) says, 'Inflammation des vaisseaux lymphatiques est le plus souvent accompagnée d'un œdème considérable des parties situées au dessous des points ou les rameaux malades se rencontret. Toutefois un engorgement spécial en est quelquefois l'effet. Le membre se tuméfie uniformément, devient tendu et luisant; des abscès s'y développent dans certains cas, et des indurations chroniques y on lieu dans d'autres circonstances.' The description which Piorry has given of lymphangioitis in general corresponds at the same time with that of phlegmatia, and hence it becomes evident that this disease is not confined to puerperal women: that it neither requires derangements of the milk or of the lochia to be induced, and that it can also occur in the male sex," &c. (p. 166.)

Our limits forbid our quoting further from the author upon this subject, although his observations are exceedingly interesting and valuable. He cites several modern authorities, more especially that of Dr. v. Ammon, in confirmation of his view that phlegmatia dolens is the result of lymphatic inflammation; we ourselves have for some time adopted this opinion, experience having fully proved to us that the notion of its identity with crural phlebitis was quite untenable.

The author's description of the erysipelatous inflammations in puerperal women is interesting, and shows both extensive observation as well as reading. Among the various appearances which the skin presents in puerperal fever, he mentions the varioloid, scarlatinoid and urticaria-like eruptions which have been occasionally observed; and we have ourselves noticed an epidemic of peculiar malignity, which occurred some years ago in this metropolis, where, but for its livid colour, the eruption bore a very close resemblance to rubeola. We quote the following passage not merely for the purpose of pointing out a peculiar appearance which is sometimes seen in these cases, but also to show how, in spite of the author's notions respecting the inflammatory origin of puerperal fever, the real nature of this disease forces itself upon him:

"I have still another appearance to mention, which seems to have less connexion with phlebitis than with the morbid state of the blood (blutdyscrasie) which arises at the same time; I allude to those circumscribed, usually bluish-red swellings, of about the size of a sixpence,* on the back of the hand and instep, or also on other parts of puerperal patients, which are usually intensely painful, and are the forerunners of approaching death. Where the patient is much reduced, there is no discoloration of the skin, and the painful swelling remains pale: the more malignant and acute the form, the greater the extent and intensity of the colour of these spots; thus in one case I saw more than half the lower extremities thus covered, this was also attended with an eruption like ecthyema, which ran through its stages very quickly." (p. 173.)

The author's observations on the treatment of phlebitis are practical and good. He appears to have used ice in different forms of puerperal inflammation pretty extensively; and as we have on former occasions called the attention of our readers to the use of this remedy, as recommended by Professor Michaelis, of Kiel, we are the more desirous to furnish whatever knowledge further experience may have gained upon the subject. Dr. Kiwisch's observations, however, refer only to the external application of ice in local puerperal inflammations, and not to the internal exhibition of it in the adynamic form of puerperal fever:

"It is especially in cases of external phlebitis, in encephalitis, the various external metastases, inflammation of the joints and phlegmatia dolens, which may be advantageously combated by the external application of ice. The development of heat over an inflamed vein is often very violent, so that the applications of ice must be frequently renewed, and applied over a considerable extent of surface in order to produce any effect. The same holds good, more or less, with encephalitis, where it is best to cover the shaven scalp with ice in bladders. In crural phlebitis and in phlegmatia, the whole extremity must be enveloped in ice fomentations, and the same holds good with joints which are attacked with the arthritic inflammation. We ought not to be deterred from the external application of ice from the fear of checking either the lochia or the cutaneous perspiration, because both of these are already more or less deranged by the morbid process which is going on, and this state can only be removed by the annihilation of the disease itself. We have moreover seen the best results from the use of ice, without any of the dreaded symptoms from the suppression of the above-mentioned excretions." (p. 179.)

* The author's words are "the size of a kreutzer," which is a very vague expression; and it might either be a silver kreutzer, which is not larger than a threepenny piece, or a copper one, of about the size of a shilling.

In the chapter on inflammation of the mucous membrane of the uterus, the author gives us the choice of two names for this affection, viz. "Metrhymenitis" and "Endometritis." We object to both, and any others which might be invented, for it is obviously wrong to look upon inflammation of the mucous membrane of the uterus in the puerperal state as a distinct disease; it is merely one of the many results of puerperal fever, and cannot be described separately. As far as regards the mere anatomical appearances which are seen in post-mortem examinations of the disease, there is no objection to their being classed under different heads, according to the different tissues which have been attacked; but in discussing the subject practically we cannot separately describe or consider local affections, which are subordinate and dependent upon the general one. Dr. Kiwisch's description of the changes which are seen in the mucous membrane of the uterus after fatal puerperal fever is excellent, and as such we quote it, but, let it be understood, *not* as belonging to a distinct disease:

"The inflammatory process of exudation does not always extend itself over the whole internal surface of the uterus, but is confined to circumscribed spots, usually the site of the placenta and the cervix, two portions which undergo the most severe traumatic irritation. The exudation of lymph is usually attached very firmly to the site of the placenta, and is frequently mixed with sanies, so as to closely resemble portions of placenta, for which it has been mistaken. At the cervix, which from venous engorgement is generally of a dark blue, the lymph is attached with greatest firmness to the superficial lacerations, and from the free access of air becomes of a dark green." (p. 187.)

The author has endeavoured to show that in endometritis we shall have a consecutive inflammation of other mucous membranes of a similar character, the symptoms of which will serve as a diagnostic to the uterine affection: the idea is ingenious, but can hardly be recognized as a law; for, if so, we ought to find extensive endometritis in all cases of what Dr. Ferguson has so ably described in his second form of puerperal fever, viz. fever with gastro-enteric irritation; and vice versâ, in every autopsy where well-marked endometritis has been discovered, we ought to find corresponding traces of analogous inflammation in the intestinal canal, &c.: this is certainly not borne out by English experience; in fact we seldom, perhaps never, find exudatory inflammation of the lining membrane of the uterus as a solitary affection in fatal cases of puerperal fever, but associated and combined with phlebitis, and especially with considerable changes (softening, &c.) in the muscular tissue of the uterus. In this country it has only been met with as a result of the malignant adynamic form of puerperal fever, where not a symptom of intestinal irritation has occurred during life, still less have any inflammatory traces been found after death. Still, however, we are far from wishing to state these opinions, founded upon experience in this country, in contradiction to those of the author, supported by the ample opportunities for observation which he has enjoyed at Prague; because, as we have before said, there is no doubt that puerperal fever exhibits many peculiarities and modifications in one place which are not met with at another. The anatomical characters of endometritis are not always very distinct, and have been mistaken by many for a ragged, half-dissolved state of the decidua, portions of which had been left adhering to the uterus. It is this form of the disease which, from the author's description, exhibits many features not commonly met with in this country, particularly where it extends to the mucous membrane of the vagina:

"The disease extends either directly from the uterus to the mucous membrane of the vagina, and we find its upper extremity covered by the same exudation which covered the os uteri; or which is more frequently the case, this portion of the vagina remains free, and the inflammation fixes it seat especially at the os externum, where we find a thin stratum of yellow exudation, which is easily wiped off. In more severe cases, especially in the dysenteric form, the inflammation attacks the whole mucous membrane; the layer of exudation is mostly very firmly adherent in these cases, and infiltrated into the membrane. The upper layer of lymph is dark green, and mixed with fatty flocculi, which give the vagina a gangrenous appearance. At a later stage the exudation is thrown off, either with the whole mucous membrane, or only with its epithelium, and the raw surface is covered with delicate granulations. A more frequent result than this is acute ulceration of the mucous membrane of the vagina, especially of the posterior commissure. Abscesses form here, of variable depth, and with sharply defined edges, destroying the posterior commissure, and spreading in all directions from the lower angle of the vulva, and varying in their mode of progress according to the character of the constitutional symptoms: thus, in some cases they assume a simple inflammatory appearance, occasionally of a triangular form, from one to two inches in extent upon the posterior wall of the vagina; in others they are of a sphacelous or phagedenic character, in which case they not unusually entirely destroy the whole perineum, the external genitals, and a large portion of the vagina and urethra; they are surrounded with everted erysipelatous edges, and are covered with masses of gangrenous slough." (p. 194.)

In terminating our observations on Dr. Kiwisch's work, we sincerely regret that his attention has been so exclusively directed towards the anatomical characters of the varied and numerous effects which puerperal fever produces in different parts of the body, for he has thus prevented himself from taking a more enlarged and strictly pathological view of this formidable malady. If instead of following the superficial reasoning of those authors, who because they have found marks of inflammatory action in certain tissues accompanying puerperal fever, have therefore pronounced *the disease* to be inflammation of these structures, he had pursued the track of those pathologists who have so successfully referred this disease to its proper place among the affections produced by a vitiated condition of the blood, he would have taken a more extended view of the subject, and attained much more correct and philosophical deductions respecting its habitudes and varieties: he would not only have given a better enumeration of its symptoms, but probably have been led to adopt more efficient means of treatment. Instead of confining himself to bleeding, purgatives, tartar emetic, small salivating doses of mercury, and oleaginous medicines, remedies which in the adynamic form of puerperal fever are not only inefficient, but sometimes even inadmissible, he would have tried the effects of occasional large doses of calomel as recommended by Dr. Ferguson, and also of saline medicines, which according to our experience are two of the most valuable means which we have to rely upon.

The value of the cases at the end of the work is much deteriorated by the author's preconceived opinions; for under the head of Metrhymenitis he brings a number of different affections, simply because there has been more or less derangement of the intestinal mucous membrane.

The idea that putrid matter in the uterus can be absorbed into the system does not once seem to strike him; and even where he does refer to a vitiated state of blood (blut-dyscrasie) his notions are vague and imperfect. Nos. 5 and 14 of his cases are, in our opinion, evidently produced by the presence of putrid matter in the uterus and vagina, and we cannot help attributing the two relapses which occurred in No. 5 to his not having removed the offending cause by warm-water injections. There are, however, several features in these cases which show a somewhat different type to the puerperal fever usually seen in the English metropolis: the attacks are usually very early after labour, the pulse is neither so quick nor so feeble, being seldom above 100, and in many instances bearing bleeding and active purging well.

In passing our opinion upon this work, we have spared neither praise nor blame, dispensing the one as freely as the other, according to our views of the subject. Although containing so much valuable matter, based on ample experience, we cannot consider that the author has even kept pace with the knowledge of this disease, as it at present stands in this country; we therefore regret that in giving so ample a list of the works upon puerperal fever, he does not appear to have seen the valuable monograph by Dr. Ferguson to which we have so often alluded.

---

